# Metabolic syndrome alters relationships between cardiometabolic variables, cognition and white matter hyperintensity load

**DOI:** 10.1038/s41598-019-40630-6

**Published:** 2019-03-13

**Authors:** E. Alkan, T. P. Taporoski, A. Sterr, M. von Schantz, H. Vallada, J. E. Krieger, A. C. Pereira, R. Alvim, A. R. V. R. Horimoto, S. Pompéia, A. B. Negrão, S. L. H. Evans

**Affiliations:** 1 Faculty of Health and Medical Sciences, University of Surrey, Guildford, Surrey United Kingdom; 2 Laboratory of Genetics and Molecular Cardiology, Heart Institute (Incor), University of São Paulo Medical School, São Paulo, Brazil; 3 Institute of Psychiatry, University of São Paulo Medical School, São Paulo, Brazil; 4 Departamento de Psicobiologia, Universidade Federal de São Paulo, São Paulo, Brazil

## Abstract

Cardiometabolic risk factors influence white matter hyperintensity (WMH) development: in metabolic syndrome (MetS), higher WMH load is often reported but the relationships between specific cardiometabolic variables, WMH load and cognitive performance are uncertain. We investigated these in a Brazilian sample (aged 50–85) with (N = 61) and without (N = 103) MetS. Stepwise regression models identified effects of cardiometabolic and demographic variables on WMH load (from FLAIR MRI) and verbal recall performance. WMH volume was greater in MetS, but verbal recall performance was not impaired. Age showed the strongest relationship with WMH load. Across all participants, systolic blood pressure (SBP) and fasting blood glucose were also contributors, and WMH volume was negatively associated with verbal recall performance. In non-MetS, higher HbA1c, SBP, and number of MetS components were linked to poorer recall performance while higher triglyceride levels appeared to be protective. In MetS only, these relationships were absent but education exerted a strongly protective effect on recall performance. Thus, results support MetS as a construct: the clustering of cardiometabolic variables in MetS alters their individual relationships with cognition; instead, MetS is characterised by a greater reliance on cognitive reserve mechanisms. In non-MetS, strategies to control HbA1c and SBP should be prioritised as these have the largest impact on cognition.

## Introduction

White matter hyperintensities (WMH) are a common finding in older adult populations, appearing as bright spots on fluid-attenuated inversion recovery (FLAIR) structural MRI acquisitions. The primary causal factor for development of WMH appears to be pathology of the brain’s small blood vessels^1^. Chronic ischaemia due to arteriolosclerosis has been linked to WMH accumulation in post-mortem studies^2,3^; transient ischemia resulting from impaired cerebrovascular autoregulation might also contribute, particularly in hypertension^4,5^, while neurovascular pathology might itself trigger compensatory changes in peripheral cardiometabolic variables via neuroregulatory mechanisms^6^. These lines of evidence suggest that WMH might be a useful clinical marker representing the interaction between cardiometabolic health and neural integrity.

WMH prevalence has a clear relationship with age. Individuals aged 55–65 possess a tenfold greater WMH load compared to those aged <55^7^; by age 70, brain WMH volume increases by around 13% per year, although considerable individual variation exists^8^. Consistent with the pathological processes thought to underlie WMH, imaging studies show that cardiometabolic variables link to WMH burden. Hypertension has been shown to be predictive of WMH progression and severity in older adults^9–11^ although not all these longitudinal studies considered other cardiometabolic variables. This is important since other factors have been linked to WMH burden in cross-sectional studies (e.g. poorer glycaemic control as indicated by elevated levels of glycated haemoglobin A1c (HbA1c)^12,13^). In the current study, we aimed to clarify these relationships with WMH load, by considering a comprehensive range of relevant variables. Including a wide range of variables is important so as to support causal inferences; strong claims regarding causality can be problematic since even longitudinal studies are open to alternative interpretations.

We also investigated whether these relationships are altered in individuals with metabolic syndrome (MetS). MetS involves a ‘clustering’ of cardiometabolic factors; MetS diagnosis requires meeting criteria on at least 3 of the 5 ‘components’ of MetS (abdominal obesity, hypertension, high blood sugar, high serum triglycerides and low high-density lipoprotein (HDL) levels). Studies have shown WMH load to be higher in MetS. In a sample aged 28–78 (mean age 53), MetS was linked to WMH prevalence with an OR = 3.3: of the MetS components, elevated blood pressure (BP) was the strongest risk factor while high fasting glucose and elevated triglycerides were also independent contributors^14^. Choi *et al*.^15^ used a larger sample with a similar mean age but found that hypertension was as good a predictor as MetS in explaining WMH prevalence. However, in an older population (mean age 71), MetS increased the risk of presenting with high levels of WMH volume (OR = 2.74) and the MetS–WMH relationship remained significant even after adjustment for hypertension^16^. Thus, although MetS has been consistently linked to WMH load, the contributions of individual cardiometabolic variables and the explanatory value of a MetS diagnosis over and above these is not clear.

Cardiometabolic variables likely interact in complex ways to influence WMH severity; a large study in older stroke patients found that in hypertensives, age and raised cholesterol showed a negative relationship with WMH burden, while diabetes mellitus and hyperlipidaemia showed a positive relationship; in non-hypertensives, only age was significant^17^. Thus we hypothesised that in MetS, the clustering of cardiometabolic factors would affect their individual relationships with WMH load. Relationships between cardiometabolic variables (including those relevant for MetS diagnosis, also low density lipoproteins, triglycerides, total cholesterol and HbA1c) and WMH load were therefore assessed and contrasted between MetS and non-MetS subgroups. Identifying differences between subgroups could inform tailored intervention strategies to minimise WMH accumulation (i.e. which factors should be targeted in MetS vs. non-MetS); if differences are identified it would also support the concept of MetS as a useful diagnosis with distinct clinical consequences, which some authors have questioned^18^.

Large longitudinal studies have suggested that WMH load contributes to future risk of cognitive decline, mild cognitive impairment^19^ and dementia^20^, while cross sectional studies in healthy populations have shown WMH load to have a small but consistent negative impact on cognitive performance^21^. Cognitive impairment and greater cognitive decline has also been observed in MetS^22,23^: some studies have identified executive function impairments in MetS and linked these to poor glycaemic control in particular^24–27^. However, the literature is inconsistent since other studies report no cognitive performance differences^28,29^; some studies in elderly and the oldest old have even found improved cognitive performance^30^ and decreased cognitive decline in MetS^31^. Thus, the associations between MetS, individual cardiometabolic factors, and cognition requires further investigation; WMH load in MetS could contribute to reports of poorer cognition, explaining some of these inconsistencies in this literature. In the present study we investigated the extent to which WMH load, cardiometabolic health, and years of education influence cognition in MetS patients as compared to non-MetS.

In contrast to some previous studies, we focused on a 50–85 age range, on the grounds that WMH burden tends to be minimal before the 5^th^ decade of life. Participants were drawn from the Baependi heart study^32^, an ongoing cohort study in a small town in rural Brazil. Baependi comprises both an ‘urban’ and ‘rural’ zone; high levels of variance in education levels are a particular strength of this dataset. WMH load was calculated from FLAIR images using a well-validated automated segmentation algorithm^33–35^ which outputs number and total volume of WMH as continuous variables; we entered these into our statistical models. This offers an advantage over some previous studies using manual raters, which relied on categorical measures, simply classifying WMH as being present or absent (e.g. Choi *et al*.^15^) or according to 3 levels of severity (e.g. Park *et al*.^14^).

## Methods

### Study population

The Baependi Heart Study began in 2005 as a longitudinal cohort study to evaluate environmental and genetic effects on cardiovascular disease risk factors^36^. Baependi is a town in a rural area (752 km^2^, 18 307 inhabitants at the 2010 census) located in the Southeast of Brazil. It is a traditional community, with a cohesive culture, a high degree of admixture and very limited inbound migration. In this study, we conducted a cross-sectional analysis of data collected in the second wave of data collection which took place between 2010 and 2015. MRI acquisitions have only been conducted for a subset of study participants due to resource limitations. The full study sample consists of 1717 subjects aged between 18 and 91 (mean age 46.59). For the purposes of the present work, inclusion criteria were: age 50–85; a complete set of cardiometabolic measures available; MRI scan completed and no image artefacts present (all MRI images were visually inspected for quality and consistency). Of these 1717, 164 individuals met these criteria and were included in the present analysis. Compared to the full study sample, this sample had a significantly lower proportion of current smokers (11.8%, compared to 17.6% in the full sample, χ² test, p < 0.001), higher mean age (p < 0.001), lower levels of education (6.30 yrs, compared to 7.61 yrs in the full sample, p < 0.001), higher triglyceride, HbA1c, and fasting blood glucose, and higher waist circumference, SBP and DBP (p < 0.05); there were no differences in HDL-C levels (t-tests).The mean interval between cardiometabolic assessment and MRI was approximately 3 yrs. The study protocol was approved by the ethics committee of the Hospital das Clínicas, University of São Paulo, Brazil, and each subject provided informed written consent before participation. All experimental procedures were performed in accordance with relevant guidelines and regulations. The sampling methodology and additional characteristics of the participants have been defined previously^32^.

### Measures

Smoking status (current, former, never) and level of education in years were recorded; smoking status data was not available for 11 participants. Height and waist circumference were measured in centimetres and weight in kilograms using a calibrated digital balance. Body mass index (BMI) was calculated as body weight (kg) divided by height squared (m^2^). BP was measured using a standard digital sphygmomanometer (OMRON, Kyoto, Japan) on the left arm after 5 min rest, in the sitting position. Systolic (SBP) and diastolic blood pressures (DBP) were calculated by taking the mean of three readings (minimum interval of 3 min between readings). High and low density lipoproteins, triglycerides, total cholesterol and fasting glucose were evaluated by standard techniques in 12-h fasting blood samples. HbA1c levels were determined by high-performance liquid chromatography (HPLC).

### Definition of MetS

MetS was defined according to National Cholesterol Education Program–Adult Treatment Panel III criteria^37^. MetS diagnosis was based on meeting criteria for 3 or more of the following 5 components: (a) waist circumference: >102 cm (men)/>88 cm (women); (b) serum triglycerides: ≥1.7 mM/L; (c) high-density lipoprotein cholesterol (HDL-C): <1.04 mM/L (men)/<1.29 mM/L (women); (d) systolic/diastolic BP: ≥130 mm Hg/≥85 mm Hg or use of antihypertensive drugs; (e) fasting blood glucose: ≥6.1 mM/L or presence of type 2 diabetes or diabetic treatment.

### Cognitive testing-Word List Recall task

The memory task was drawn from the brief neuropsychological battery developed by The Consortium to Establish a Registry for Alzheimer’s disease (CERAD); previous work has established their suitability for use in Brazilian populations^38^. *Task procedure:* 10 unrelated items are presented to participants one at a time on printed cards, and they are instructed to read aloud each item as it is presented. Immediately after all 10 have been presented, the participant is asked to recall as many items as possible. There are 3 learning trials, with the same 10 words presented but in a different order. The maximum score on each trial is 10. The maximum total score is 30. Here, we considered three measures from this task. Word List: Total (recall performance summed across all 3 trials), Word List: Immediate (recall performance on the first trial only), and Word List: Learning (improvement in recall performance across trials: words recalled on trial 3 minus words recalled on trial 1).

### MRI acquisition

MRI scans were obtained at the Hospital Cônego Monte Raso in Baependi on a 1.5 T MAGNETOM (Siemens, Munich, Germany). High-resolution T1 images were acquired using a three-dimensional fast spoiled gradient echo T1-weighted sequence (Voxel size 1 mm^3^, 160 slices, Matrix Size 256 × 256, TR 1700ms, TE 5.1 ms, flip angle 120, inversion time 850 ms). A T2-weighted FLAIR sequence (Matrix Size 280 × 320, Voxel size 0.719 × 0.719 × 6.32 mm, axial slice width 5.5 mm with 0.82 mm interslice gap, 20 slices, TR 1000.2 ms, TE 109.3 ms, flip angle 150, inversion time 2500 ms) was acquired.

### WMH Segmentation

Automatic segmentation of WMHs was performed using the lesion segmentation toolbox (LST)^35^ (http://www.applied-statistics.de/lst.htm) implemented within SPM12. Previous studies that investigated effects of cardiometabolic variables on WMH volume using the LST guided our methodology^34,39^. The lesion growth algorithm (LGA) was used in the LST with the following parameters: binary = 0.50, kappa = 0.25, based on a previous study that determined an optimal kappa of 0.25 to maximise consistency with manual raters, in participants with diabetes^34^. The LGA first segments the T1 image, these are then combined with the coregistered FLAIR intensities in order to calculate lesion belief maps. These are then thresholded at the selected kappa to create a binary lesion map which is subsequently grown along voxels that appear hyperintense in the FLAIR image. The result is a lesion probability map. Number of discrete WMH, and total WMH volume (ml) are calculated from this. LST outputs have been shown to have high agreement with those of experienced manual raters^33,34^.

### Statistical Analysis

MetS/non-MetS subgroups were compared using t-tests (for continuous variables), Pearson’s χ2 test (for categorical variables). ANOVA was used to assess effects of gender and smoking status on WMH volume and number, and interaction with MetS diagnosis. As a preliminary analysis, a series of linear regressions were conducted to assess the relationships between WMH volume, WMH number, number of MetS components present, years of education, and age, as well as the cognitive performance scores from the word list (WL) task: Total, Immediate and Learning.

Step-wise regression models were then constructed. For the WMH measures, WMH volume and WMH number were the dependent variables (separate models); age, education, BMI, waist circumference, total cholesterol, fasting blood glucose, triglyceride, HDL-C, LDL-C, systolic BP, diastolic BP, and number of MetS components were entered as independent variables. For the cognitive measures, word list (WL): Total, Immediate and Learning scores were the dependent variables (separate models); age, education, BMI, waist circumference, total cholesterol, fasting blood glucose, triglyceride, HDL-C, LDL-C, systolic BP, diastolic BP, number of MetS components, WMH volume, and WMH number were entered as independent variables.

All statistical tests were implemented in SPSS version 24.0 for Windows (SPSS Inc., Chicago, IL), p values are reported two tailed and p < 0.05 was considered statistically significant. To assess multicollinearity between variables in each of the models, the variation inflation factor (VIF) was calculated for each variable. Multicollinearity was observed to be low: the VIF values never approached or exceeded the generally-accepted critical value of 10, and in fact were typically much lower (<3).

## Results

### Sample characteristics

This study comprised 164 individuals (59.1% female) aged 50–85 (mean age 60.09, SD 7.89). Of these, 61 (37%) met criteria for MetS diagnosis. The characteristics of all participants, and the MetS subgroup, is shown in Table 1. The MetS subgroup did not differ in age, gender, smoking status, socioeconomic status and years of education. Subjects with MetS had significantly higher body mass index, waist circumference, systolic BP, fasting blood glucose, total cholesterol, triglyceride and HbA1c levels, and lower HDL-C levels. However, there were no statistically significant differences between subgroups in LDL-C level and diastolic BP. For all components of MetS, proportion of subjects meeting criterion for each component was higher in the MetS subgroup, namely elevated BP (63.9%), elevated fasting blood glucose (37.7%), elevated triglyceride (82.0%), low HDL-C (85.2%) and abdominal obesity (72.1%). The MetS subgroup had significantly higher WMH volume (p = 0.034), but not WMH number. ANOVA revealed no effect of gender on WMH volume (F_1,163_ = 0.000, p = 0.997) or number (F_1,163_ = 0.178, p = 0.673), and no interactions with MetS diagnosis. Similarly, smoking status did not impact WMH volume (F_1,152_ = 0.683, p = 0.507), or number (F_1,152_ = 1.255, p = 0.288), and there were no interactions with MetS diagnosis. Verbal recall performance (word list: total, immediate, learning) showed no significant differences between subgroups.

**Table 1:** Participant characteristics (All, non-MetS, MetS), *p* values from t-tests comparing subgroups (non-MetS vs. MetS).

	All (*n* = 164)	Non-MetS (*n* = 103)	MetS (*n* = 61)	*p*
Age, M (SD)	60.09 ± 7.89	59.70 ± 7.82	60.75 ± 8.03	0.409
Female (N, %)	97 (59.1%)	58 (56.3%)	39 (63.9%)	0.337
Education (years)	6.30 ± 4.40	6.40 ± 4.56	6.15 ± 4.16	0.726
Socioeconomic status	1.92 ± 0.53	1.92 ± 0.63	1.91 ± 0.29	0.883
Body Mass Index (kg/m^2^)	27.18 ± 4.66	26.28 ± 4.74	28.69 ± 4.13	<0.001
Waist Circumference (cm)	95.26 ± 11.07	93.02 ± 11.08	99.03 ± 10.05	<0.001
Systolic BP (mmHg)	129.60 ± 16.88	125.59 ± 14.70	136.36 ± 18.23	<0.001
Diastolic BP (mmHg)	79.53 ± 19.16	78.41 ± 23.18	81.43 ± 8.85	0.331
Fasting Blood Glucose (mM)	5.62 ± 1.9	5.10 ± 1.0	6.40 ± 2.7	<0.001
Total Cholesterol (mM)	5.41 ± 1.1	5.25 ± 0.9	5.66 ± 1.10	0.013
Triglyceride (mM)	1.72 ± 1.0	1.35 ± 0.6	2.39 ± 1.2	<0.001
HDL-C (mM)	1.21 ± 0.3	1.27 ± 0.3	1.11 ± 0.2	<0.001
LDL-C (mM)	3.40 ± 0.9	3.36 ± 0.8	3.49 ± 1.0	0.353
HbA1c	6.07 ± 1.2	5.80 ± 0.7	6.53 ± 1.69	<0.001
Smokers (N, %) (Current Smokers)	18 (11.8%)	11 (11.5%)	7 (12.3%)	0.979
**MetS Components:**				
Elevated BP	69 (54.5%)	30 (29.1%)	39 (63.9%)	<0.001
Elevated Fasting Blood Glucose	29 (17.7%)	6 (5.8%)	23 (37.7%)	<0.001
Elevated Triglyceride	68 (41.5%)	18 (17.5%)	50 (82.0%)	<0.001
Low HDL-C	90 (54.9%)	38 (36.9%)	52 (85.2%)	<0.001
Abdominal Obesity	85 (53.4%)	41 (39.8%)	44 (72.1%)	<0.001
**WMH:** Volume (ml), M (SD)	0.98 ± 1.9	0.73 ± 1.3	1.39 ± 2.6	0.034
**WMH:** Number, M (SD)	4.86 ± 4.6	4.43 ± 4.0	5.59 ± 5.6	0.122
**Word List:** Total	18.04 ± 4.1	18.15 ± 4.2	17.9 ± 3.9	0.669
**Word List:** Immediate	4.37 ± 1.5	4.44 ± 1.6	4.28 ± 1.4	0.527
**Word List:** Learning	3.08 ± 1.6	2.96 ± 1.7	3.28 ± 1.4	0.225

Abbreviations: HbA1c, Haemoglobin A1c; HDL-C, high density lipoprotein cholesterol; LDL-C, low density lipoprotein cholesterol; WMH, White Matter Hyperintensities; M (SD), mean (standard deviation).

### Linear regression analyses

#### All subjects

Age was positively correlated with WMH volume (r = 0.422, n = 164, p < 0.001), as was WMH number (r = 0.454, p = < 0.001). Number of components was significantly correlated with WMH volume (r = 0.186, p = 0.017) and WMH number (r = 0.175, p = 0.025). WMH volume negatively correlated with WL:Total (r = −0.166, p = 0.034). Years of education was positively correlated with WL: learning scores (r = 0.197, p = 0.011), see Table 2.

**Table 2:** Linear regression analyses for all subjects, and non-MetS, MetS subgroups.

	WMH Volume			WMH Number			WL: Total			WL: Immediate			WL: Learning		
	All	Non- Mets	With MetS	All	Non- Mets	With MetS	All	Non- Mets	With MetS	All	Non- Mets	With MetS	All	Non- Mets	With MetS
WMH Volume	—	—	—	0.797**	0.774**	0.822**	−0.166*	−0.195*	−0.154	−0.120	−0.166	−0.080	−0.081	−0.060	−0.164
WMH Number	—	—	—	—	—	—	−0.086	−0.100	−0.064	−0.069	−0.076	−0.050	−0.056	−0.070	−0.074
No. of Components	0.186*	0.035	0.157	0.175*	0.034	0.285*	−0.106	−0.178	−0.051	−0.115	−0.123	−0.145	0.109	−0.006	0.209
Years of education	−0.142	−0.141	−0.157	−0.079	−0.036	−0.133	0.101	0.114	0.074	−0.004	0.063	−0.150	0.197*	0.125	0.383**
Age	0.422**	0.466**	0.417**	0.454**	0.446**	0.467**	−0.111	−0.105	−0.117	−0.066	−0.110	0.024	−0.094	−0.238	−0.235

Note —Data are Pearson correlation coefficients (r). *Abbreviations*: WMH White Matter Hyperintensities, MetS Metabolic Syndrome **p < 0.01. *p < 05.

#### Analyses by group (non-MetS, MetS)

Correlations were then assessed separately for the MetS and non- MetS subgroups. This subgroup analysis revealed some differences between groups: only in MetS did the number of components positively correlate with WMH number (r = 0.285, n = 61, p = 0.017), while only in non-MetS was a negative correlation seen between WMH volume and WL:Total scores (r = −0.195, n = 103, p = 0.011). The relationship between years of education and WL:Learning scores was only significant in MetS (r = 0.383, n = 61, p = 0.002).

### Multiple regression analyses

A series of multiple regression models were constructed to determine the predictive value of individual cardiometabolic variables, education, and age on WMH number and volume, and verbal recall. Stepwise multiple regression was employed. A summary of significant predictors are presented in Table 3. Full statistical outputs from the regression models are reported in the supplementary materials. Models were built to assess effects in all subjects, then separately for the non-MetS and MetS groups.

**Table 3:** Variables explaining significant variance, identified by the stepwise multiple regression models, constructed separately for all subjects, MetS and non-MetS subgroups.

Dependent Variable	All Subjects	With MetS	Without MetS
WMH Volume	Age (R² = 0.178, β = 0.359, p = 0.000) SBP (∆R² = 0.038, β = 0.206, p = 0.006)	Age (R² = 0.173, β = 0.417, p = 0.000)	Age (R² = 0.217, β = 0.466, p = 0.000)
WMH Number	Age (R² = 0.206, β = 0.433, p = 0.000) Fasting Blood Glucose (∆R² = 0.034, β = 0.185, p = 0.034)	Age (R² = 0.218, β = 0.467, p = 0.000)	Age (R² = 0.199, β = 0.446, p = 0.000)
Word List: Total	WMH volume (R² = 0.027, β = −0.166, p = 0.000)	None	HbA1c (R² = 0.038, β = −0.178, p = 0.050) Triglyceride (∆R² = 0.045, β = 0.275, p = 0.030) Number of MetS Components (∆R² = 0.042, β = −0.220, p = 0.032)
Word List: Immediate	HbA1c (R² = 0.031, β = −0.175, p = 0.027)	None	None
Word List: Learning	Education (R² = 0.039, β = 0.197, p = 0.012)	Education (R² = 0.146, β = 0.383, p = 0.003)	Triglyceride (R² = 0.041, β = 0.195, p = 0.040) SBP (∆R² = 0.038, β = −0.193, p = 0.048)

SBP, Systolic BP.

### All Subjects

#### WMH volume and number

SBP and age were significant predictors of WMH volume. These two variables together explained about 22% of the total variance in WMH volume (R = 0.465, R² = 0.216, F _(1,159)_ = 21.690, p = 0.000): age explained about 18% of the variance (R = 0.422, R² = 0.178, F _(1, 158)_ = 34.247, p = 0.000 β = 0.359) while SBP contributed the remaining 4% (ΔR² = 0.038, F _(1, 157)_ = 7.685, p = 0.006 β = 0.206). Number of components (p = 0.212), DBP (p = 0.980), BMI (p = 0.127), waist circumference (p = 0.153), HbA1c (p = 0.156), triglyceride (p = 0.358), fasting blood glucose (p = 0.090), HDL-C (p = 0.504) and LDL-c (p = 0.156) did not significantly predict WMH volume.

Fasting blood glucose and age were significant predictors of WMH number. These two variables together explained about 24% of the total variance in WMH number (R = 0.490, R² = 0.240, F _(1, 158)_ = 24.786, p = 0.000, β = 0.433). Again, age explained the majority of the variance, while fasting blood glucose contributed to the total variance at 3% (ΔR² = 0.034, F _(1, 157)_ = 6.984, p = 0.034, β = 0.185).

SBP (p = 0.097), DBP (p = 0.986), BMI (p = 0.247), waist circumference (p = 0.173), HbA1c (p = 0.093), triglyceride (p = 0.756), total cholesterol (p = 0.544), number of components (p = 0.285) HDL-C (p = 0.703) and LDL-c (p = 0.756) did not significantly predict WMH number.

#### Cognitive measures

WMH volume was the only predictor of WL total scores and explained about 3% of the variance in performance (R = 0.166, R² = 0.021, F _(1, 158)_ = 4.468, p = 0.036 β = −0.166). HbA1c was the only predictor of WL immediate recall, explaining about 3% of the variance (R = 0.175, R² = 0.031, F _(1, 158)_ = 4.987, p = 0.027(β = −0.175), with higher HbA1c levels linked to lower performance. Education was the only predictor of learning, explaining about 4% of the variance (R = 0.197, R² = 0.039, F _(1, 158)_ = 6.410, p = 0.012, β = 0.197).

### Analyses by group (non-MetS, MetS)

#### WMH volume and number

In non-MetS subjects, age was the only predictor of WMH volume (R = 0.466, R² = 0.217, F _(1, 100)_ = 27.772, p = 0.000, β = 0.466) and number (R = 0.446, R² = 0.199, F _(1, 100)_ = 24.837, p = 0.000, β = 0.446), explaining about 22% and 20% of the variance respectively.

Similarly, in MetS subjects, age was the only predictor of WMH volume (R = 0.417, R² = 0.173, F _(1, 58)_ = 11.754, p = 0.000, β = 0.417) and number (R = 0.467, R² = 0.218, F _(1, 56)_ = 15.654, p = 0.000, β = 0.467), explaining about 17% and 22% of the variance respectively.

#### Cognitive Measures

In subjects without MetS, it was found that HbA1c (R = 0.195, R² = 0.038, F _(1, 100)_ = 3.944, p = 0.050, β = −0.195), triglyceride (ΔR² = 0.45, F _(1, 99)_ = 4.830, p = 0.014, β = 0.213) and number of components (ΔR² = 0.42, F _(1, 98)_ = 4.716, p = 0.032, β = −0.396) were significant predictors of WL: Total scores. Higher HbA1c and number of components negatively impacted WL: Total performance, but higher triglyceride was associated with better scores, suggesting a protective role for triglyceride on this measure. In total, the model explained 12.5% of variance in WL:Total scores, with each of the three predictors contributing ~4%. There were no significant predictors for WL:Immediate scores, but triglyceride (R = 0.203, R² = 0.041, F _(1, 100)_ = 4.318, p = 0.040, β = 0.193) and SBP (ΔR² = 0.37, F _(1, 99)_ = 4.226, p = 0.048, β = −0.193) were significant predictors of WL:Learning scores in non- MetS. Again, triglyceride levels emerged as a protective factor in learning performance, with triglyceride and SBP each independently explaining about 4% of the variance in learning scores.

In subjects with MetS there were no significant predictors for WL:Total scores and WL:Immediate scores. Education significantly predicted learning scores, explaining about 15% of the variance (R = 0.383, R² = 0.146, F _(1, 56)_ = 9.609, p = 0.003, β = 0.383).

## Discussion

In a sample of 164 individuals aged 50–85 we conducted analyses to investigate how cardiometabolic and demographic factors influence WMH load, and how these relate to cognitive performance using a word list recall task. Linear and multiple regression models were employed, firstly across all subjects, and then separately for subjects with and without MetS. 37% of participants met criteria for MetS, and MetS/non-MetS subgroups were well matched in age, gender balance, education and smoking status. The MetS subgroup had a significantly greater WMH load in terms of volume (but not number), consistent with the literature^14–16^. However, no significant difference between subgroups was observed on any of the verbal recall measures. Evidence for a cognitive deficit in MetS is inconsistent^22,23^. For example, while Alfaro *et al*.^24^ reported a deficit in verbal learning, Sala *et al*.^28^ found no differences in picture learning performance; both studies had a mean age of 65. A larger study (n = 3369) in males of mean age 60 (similar to the present study) reported no effects of MetS on memory^29^. The findings of the present study therefore strengthen the idea that the metabolic syndrome is not reliably associated with cognitive deficits. However, the cognitive test used in the present study provides a limited assessment of cognitive performance which may not have been sensitive enough to identify more subtle deficits.

Initial analyses investigated correlations between variables. As expected, age was strongly correlated with WMH number and volume. The number of MetS components participants met criteria for, correlated positively with WMH number and volume (but not when MetS/non-MetS subgroups were analysed separately). There was a negative impact of WMH volume on verbal recall (particularly in non-MetS individuals). More years of education was positively correlated with learning on the verbal recall task, but in MetS only. Follow-up multiple regression analyses explored the data in more depth. Individual cardiometabolic and demographic variables were assessed as independent predictors of WMH load and verbal recall performance, by subgroup.

Consistent with the regression analyses, the multiple regression models pointed to age as having the most significant relationship with WMH load, explaining ~18% of the variance in volume, and ~20% of the variance in number. Across all subjects, significant contributions from cardiometabolic variables were also identified. Specifically, SBP explained an additional 4% of variance in WMH volume, fasting blood glucose level an additional 4% of variance in WMH number. Previous work has also identified effects of SBP. Griffanti, *et al*.^40^ found that SBP correlated with periventricular WMH load in healthy adults aged 60–83. A study in healthy elderly found that for each 1 mm Hg increase of systolic BP, OR of moderate vs. mild WMH severity degree increased by more than 1%^41^. However, both these studies considered only a limited set of cardiometabolic measures and in the case of Basile, *et al*.^41^, a coarse classification of WMH load (mild, moderate, severe) was used. The present results enhance these findings by quantifying the impact of SBP on absolute WMH volumes while considering SBP within a comprehensive array of cardiometabolic variables. Results are also consistent with longitudinal studies in older adults showing that baseline hypertension is predictive of WMH severity^9–11^. Mechanistically, this could be due to hypertension causing increases in arterial stiffness, since WMH volume has been shown to correlate with arterial stiffness after adjusting for BP in healthy individuals aged 70+^42^. Thus, higher BP could accelerate WMH formation by impairing cerebrovascular reactivity and increasing the likelihood of cerebral ischemia^5,43^. We also found a positive relationship between fasting blood glucose and WMH number. Increased fasting blood glucose has been linked previously to WMH load^44^, and particularly to WMH in frontal and temporal regions^45^. WMH in these regions tend to be smaller and discrete (thus increasing WMH *number*), as compared to those located periventricularly (which tend to be continuous and thus contributing more to overall WMH *volume*). Thus, our results accord with previous findings, and suggest that fasting blood glucose might influence WMH particularly in non-periventricular regions, while SBP links to WMH located periventricularly. Further work is needed to confirm this distinction.

The initial correlation analyses suggested a link between the number of MetS components and WMH number and volume, across all subjects. This was not confirmed in the regression models, which instead identified SBP alone as a significant predictor of WMH volume. This confirms the results of Choi *et al*.^15^ who found that a MetS diagnosis offered no additional explanatory power over hypertension in terms of WMH severity; here we show that SBP alone is a better predictor of absolute WMH volume, with number of components not emerging as a significant predictor in stepwise regression modelling. This highlights the role of SBP in WMH formation, and the importance of controlling SBP to maintain neural integrity.

When MetS and non-MetS groups were analysed separately, only age showed a significant relationship with WMH volume and number, likely due to the smaller number of participants within each of the subgroups. This highlights a weakness of stratified analyses whereby reduced statistical power can lead to a failure to detect associations (and increase the risk of false positives), underlining the need for large sample sizes in future work. Similarly, with regards to the verbal recall measures, only WMH volume was seen to explain significant variance in Word List: Total scores (recall performance across all 3 learning trials), but only when all subjects were included in the analysis, and the amount of variance explained was relatively small (~3%). Since the MetS subgroup presented with higher WMH volume but did not differ in verbal recall performance, this suggests that WMH load does not mediate cognition in this subgroup specifically; the regression models confirm this. The few previous studies to also address this directly reported similar findings. Bokura *et al*.^25^ found impaired executive function in MetS to be independent of WMH load in a sample aged 44–86, as did Viscogliosi *et al*.^46^ in an elderly population. Portet *et al*.^16^ reported higher WMH volumes in MetS despite no differences in MMSE score. Thus our results support the notion that WMH load is not a mediating factor for the cognitive impairments sometimes reported in MetS.

Across all participants, WMH volume explained variance in overall verbal recall performance. This is in line with previous studies in healthy older adults that have shown WMH to impact executive function and processing speed^40,47^, and episodic memory performance^48^, possibly by promoting cortical thinning and medial temporal lobe atrophy^49,50^.

When analysed by subgroup, some interesting differences emerged. In non-MetS participants, variance in total word recall was explained by HbA1c, in line with previous work: both in the presence and absence of MetS-related differences, higher fasting blood glucose has been shown to undermine executive function^25,29^. It should be noted that both these previous studies focussed on non-verbal measures of executive function; the current study extends these findings into the domain of verbal memory. Number of components also explained variance in non-MetS total recall, and higher SBP explained variance in non-MetS learning performance (~4% each). There is good evidence for a link between higher BP and lower cognitive function^51^, although many studies have focussed on late life and employed tests assessing general cognition only. Here we demonstrate that specifically SBP links to poorer learning across trials in a verbal recall task, in non-MetS individuals of mean age 60. Also in the non-MetS subgroup, meeting criteria on more components of MetS explained variance in overall recall performance, indicating a cumulative negative effect on cognition. A small (~4%) positive effect of higher triglycerides was also evident on total recall and learning in non-MetS. In healthy elderly, there is limited evidence that high normal plasma triglyceride might be protective against cognitive impairment^52^, possibly due to triglycerides promoting transport of ghrelin and insulin across the blood brain barrier, or triglycerides indicating superior nutritional status. However, this might not be the case in leptin-resistant conditions such as MetS^53^, further work is needed. Also, given the relatively small amounts of variance explained by each of the variables discussed in this paragraph (~4%), it should be noted that while informative from a mechanistic standpoint, the clinical significance of these effects might be limited.

In the MetS subgroup a very different pattern of relationships was observed such that none of the variables entered were seen to explain significant variance in total word list scores, or immediate recall, in this subgroup. While some studies have linked glycaemic control to cognitive performance in MetS, other work has suggested that, like here, none of the individual MetS components can explain variance in cognitive performance^46^. This supports the validity of MetS as a discrete construct, in which the clustering of cardiometabolic factors disturbs their individual relationships with cognitive function measures. However, we found education to have substantial effects in MetS patients, explaining nearly 15% of variance in learning performance, suggesting a protective effect which appeared to be quite specific to the MetS subgroup; across all subjects, education only explained 4% of variance. The neuroprotective effects of education are well established. Cognitive reserve frameworks hypothesise that educational and occupational experiences impart a reserve against pathological changes in the brain^54^, promoting compensatory processes that can be engaged to meet cognitive challenge and thus preserve cognitive performance^55^. These data suggest that individuals with MetS are relying more heavily on such processes compared to non-MetS, presumably to compensate for the neuropathological burden associated with the syndrome. This might explain why no differences in cognitive performance were seen between the subgroups, despite the presence of negative relationships between MetS components and cognition in the non-MetS subgroup.

Previous work in a UK cohort found that occupational position, but not educational level, could attenuate MetS-related cognitive decline^56^. However, a much broader range of education level is present in the cohort studied here, which includes individuals with very low levels of schooling. Work in another Brazilian cohort revealed that education was protective against the cognitive effects of lacunar infarcts^57^. The ability to capitalise on this substantial variance in sociodemographic factors is a key strength of the current study. Our findings point to education as an important mediator of cognitive performance in individuals meeting criteria for MetS, with compensatory mechanisms active in this subgroup. This could explain why cognitive deficits in MetS are inconsistently reported in the literature, and highlights the need to carefully account for education as a possible confound in such studies. In terms of follow up work, designs that incorporate repeated testing would also be beneficial, to track the influence of the variables under study on WMH progression, rather than WMH load at a single time point. Causal relationships are hard to infer on the basis of the cross sectional data presented here: careful longitudinal designs could allow for stronger casual inferences, and also allow investigation of the possibility that the neurovascular pathology thought to underlie WMH might itself be a cause for changes in peripheral cardiometabolic status. Impaired blood supply to brain likely prompts a compensatory response via neuroregulatory mechanisms, but this has not been interrogated.

In conclusion, we have shown that SBP and fasting blood glucose contribute to WMH volume and number respectively, and WMH volume explained significant variance in verbal recall performance. MetS was associated with a significantly greater WMH load but no cognitive impairment was observed. In non-MetS, specific relationships between cardiometabolic factors and verbal recall performance were identified but these were absent in MetS. However, a large positive effect of education level on recall performance was seen in MetS, suggesting intact cognitive reserve mechanisms operating to preserve cognitive function in this subgroup, at least within the age range studied here.

## Supplementary information

Supplementary Materials

## Data Availability

The datasets generated during the current study are available from the corresponding author on reasonable request.
