# Supplementary material for: Metabolic syndrome alters relationships between cardiometabolic variables, cognition and white matter hyperintensity load
Source: Sci Rep. 2019 Mar 13;9:4356. doi: 10.1038/s41598-019-40630-6 (PMC6416472; doi:10.1038/s41598-019-40630-6)
Supplement: Supplementary file 1 — Supplementary Materials [file 41598_2019_40630_MOESM1_ESM.pdf]

Metabolic syndrome alters relationships between cardiometabolic variables, cognition and white matter hyperintensity load

Alkan, E.[1], Taporoski, T.P.[2], Sterr, A.[1], von Schantz, M.[1, 3], Vallada, H.[3], Krieger, J. E [2]. Pereira, A.C.[2], Alvim, R. [2], Horimoto, A.R.V.R. [2], Pompéia, S.[4], Negrão, A.B.[2], Evans, S[1*].

[1] Faculty of Health and Medical Sciences, University of Surrey, Guildford, Surrey, United Kingdom

[2] Laboratory of Genetics and Molecular Cardiology, Heart Institute (Incor), University of São Paulo Medical School, São Paulo, Brazil

[3] Institute of Psychiatry, University of São Paulo Medical School, São Paulo, Brazil

[4] Departamento de Psicobiologia, Universidade Federal de São Paulo, São Paulo, Brazil

***Corresponding author.**

Dr. Simon Evans

email: se0016@surrey.ac.uk

phone:01483 686945

Supplementary Materials

Results of Stepwise Regression Analyses

Table S1 Variables Predicting WMH volume for All Subjects

Model	*Predictor*	*B*	*SE*	*β*	*T*	*R*	*R²*	*ΔR²*	*F*	*p*
1	Constant	-5.124	1.052		-4.870					
	Age	.102	.017	.422	5.852	.422	.178	.178	34.247**	.000
2	Constant	-7.214	1.277		-5.650					
	Age	.086	.018	.359	4.842					
	SBP	.023	.008	.206	2.772	.465	.216	.038	7.685*	.006

***p< .01.*p< .05* SBP, Systolic BP

Table S2 Variables Predicting WMH Number for All Subjects

Model	*Predictor*	*B*	*SE*	*β*	*T*	*R*	*R²*	*ΔR²*	*F*	*p*
1	Constant	-11.228	2.533		-4.433					
	Age	.268	.042	.454	6.406	.454	.206	.206	41.033**	.000
2	Constant	-13.026	2.578		-5.054					
	Age	.255	.041	.433	6.187					
	FBG	.025	.009	.185	2.643	.490	.240	.034	6.984*	.034

***p< .01.*p< .05.* FBG, Fasting Blood Glucose

Table S3 Variables Predicting WL: Total for All Subjects

Model	***Predictor***	***B***	***SE***	***B***	***t***	***R***	***R²***	***ΔR²***	***F***	***p***
1	Constant	18.403	.364		50.603					
	WMH Volume	-.360	.171	-.166	-2.114	.166	.027	.027	4.468*	.036

**p< .05.* WMH, White Matter Hyperintensities

Table S4 Variables Predicting WL:Immediate Scores for All Subjects

Model	***Predictor***	***B***	***SE***	***β***	***t***	***R***	***R²***	***ΔR²***	***F***	***p***
1	Constant	5.713	.610		9.371					
	HbA1c	-.220	.098	-.175	-2.233	.175	.031	.031	4.987*	.027

**p< .05.*

Table S5 Variables Predicting WL:Learning Scores for All Subjects

Model	***Predictor***	***B***	***SE***	***β***	***t***	***R***	***R²***	***ΔR²***	***F***	***p***
1	Constant	2.622	.220		11.919					
	Education	.073	.029	.197	2.532	.197	.039	.039	6.410*	.012

**p< .05.*

Table S6 Variables Predicting WMH Volume for Subjects with Metabolic Syndrome

Model	***Predictor***	***B***	***SE***	***β***	***t***	***R***	***R^2***	***ΔR^2***	***F***	***p***
1	Constant	-6.692	2.378		-2.815					
	Age	.133	.039	.417	3.428	.417	.173	.173	11.754**	.000

***p< .01.*

Table S7 Variables Predicting WMH Number for Subjects with Metabolic Syndrome

Model	***Predictor***	***B***	***SE***	***β***	***t***	***R***	***R^2***	***ΔR^2***	***F***	***p***
1	Constant	-14.107	5.021		-2.810					
	Age	.324	.082	.467	3.957	.467	.218	.218	15.654**	.000

***p< .01.*

Table S8 Variables Predicting WL: Learning Scores for Subjects with Metabolic Syndrome

Model	***Predictor***	***B***	***SE***	***β***	***t***	***R***	***R^2***	***ΔR^2***	***F***	***p***
1	Constant	2.504	.301		8.326					
	Education	.126	.041	.383	3.100	.383	.146	.146	9.609*	.003

***p< .01.*

Table S9 Variables Predicting WMH Volume for Subjects without Metabolic Syndrome

Model	***Predictor***	***B***	***SE***	***β***	***t***	***R***	R^2	ΔR^2	***F***	***p***
1	Constant	-3.952	.898		-4.402					
	Age	.079	.015	.466	5.270	.466	.217	.217	27.772**	.000

***p< .01.*

Table S10 Variables Predicting WMH Number for Subjects without Metabolic Syndrome

Model	***Predictor***	***B***	***SE***	***β***	***t***	***R***	R^2	ΔR^2	***F***	***p***
1	Constant	-9.121	2.742		-3.327					
	Age	.227	.046	.446	4.984	.446	.199	.199	24.837**	.000

***p< .01.*

Table S11 Variables Predicting WL: Total for Subjects without Metabolic Syndrome

Model	*Predictor*	*B*	*SE*	*β*	*t*	*R*	R^2	ΔR^2	*F*	*p*
1	Constant	24.839	3.391		7.325					
	HbA1c	-1.153	.581	-.195	-1.986	.195	.038	.038	3.944*	.050
2	Constant	23.607	3.375		6.995					
	HbA1c	-1.311	.574	-.221	-2.282					
	Triglyceride	.018	.008	.213	2.198	.288	.083	.045	4.830*	.030
3	Constant	23.111	3.321		6.959					
	HbA1c	-1.054	.576	-.178	-1.829					
	Triglyceride	.023	.008	.275	2.766					
	Number of Components	-1.249	.575	-.220	-2.172	.353	.125	.042	4.716*	.032

$*p < .05$

Table S12 Variables Predicting WL: Learning for Subjects without Metabolic Syndrome

Model	***Predictor***	***B***	***SE***	***β***	***t***	***R***	R^2	ΔR^2	***F***	***p***
1	Constant	2.122	.438		4.844					
	Triglyceride	.007	.003	.203	2.078	.203	.041	.041	4.318*	.040
2	Constant	5.034	1.518		3.316					
	Triglyceride	.007	.003	.195	2.018					
	SBP	-.023	.011	-.193	-2.001	.280	.079	.037	4.003*	.048

*$p < .05$. SBP, Systolic BP
